# Glucokinase (GCK) Mutations and Their Characterization in MODY2 Children of Southern Italy

**DOI:** 10.1371/journal.pone.0038906

**Published:** 2012-06-20

**Authors:** Marina Capuano, Carmen Maria Garcia-Herrero, Nadia Tinto, Carla Carluccio, Valentina Capobianco, Iolanda Coto, Arturo Cola, Dario Iafusco, Adriana Franzese, Adriana Zagari, Maria Angeles Navas, Lucia Sacchetti

**Affiliations:** 1 Department of Biochemistry and Medical Biotechnology, University of Naples “Federico II”, Naples, Italy; 2 Centro di Ingegneria Genetica (CEINGE) Advanced Biotechnology, s. c. a r. l., Naples, Italy; 3 Department of Biochemistry and Molecular Biology III, Faculty of Medicine, Complutense University of Madrid, Madrid, Spain; 4 Centro de Investigación Biomédica en Red de Diabetes y Enfermedades Metabólicas Asociadas (CIBERDEM), Barcelona, Spain; 5 Department of Biological Science, University of Naples “Federico II”, Naples, Italy; 6 Fondazione SDN-IRCSS (Istituto di Diagnostica Nucleare-Istituto di Ricerca e Cura a Carattere Scientifico), Naples, Italy; 7 Department of Pediatrics, Second University of Naples, Naples, Italy; 8 Department of Pediatrics, University of Naples “Federico II”, Naples, Italy; Lund University Hospital, Sweden

## Abstract

Type 2 Maturity Onset Diabetes of the Young (MODY2) is a monogenic autosomal disease characterized by a primary defect in insulin secretion and hyperglycemia. It results from GCK gene mutations that impair enzyme activity. Between 2006 and 2010, we investigated GCK mutations in 66 diabetic children from southern Italy with suspected MODY2. Denaturing High Performance Liquid Chromatography (DHPLC) and sequence analysis revealed 19 GCK mutations in 28 children, six of which were novel: p.Glu40Asp, p.Val154Leu, p.Arg447Glyfs, p.Lys458_Cys461del, p.Glu395_Arg397del and c.580-2A>T. We evaluated the effect of these 19 mutations using bioinformatic tools such as Polymorphism Phenotyping (Polyphen), Sorting Intolerant From Tolerant (SIFT) and *in silico* modelling. We also conducted a functional study to evaluate the pathogenic significance of seven mutations that are among the most severe mutations found in our population, and have never been characterized: p.Glu70Asp, p.His137Asp, p.Phe150Tyr, p.Val154Leu, p.Gly162Asp, p.Arg303Trp and p.Arg392Ser. These seven mutations, by altering one or more kinetic parameters, reduced enzyme catalytic activity by >40%. All mutations except p.Glu70Asp displayed thermal-instability, indeed >50% of enzyme activity was lost at 50°C/30 min. Thus, these seven mutations play a pathogenic role in MODY2 insurgence. In conclusion, this report revealed six novel GCK mutations and sheds some light on the structure-function relationship of human GCK mutations and MODY2.

## Introduction

Maturity Onset Diabetes of the Young (MODY), a monogenic diabetes inherited in an autosomal dominant mode, accounts for 1–2% of all diabetes forms in Europe [1], [2]. It is a clinically heterogeneous group of diseases caused by at least eight gene defects in the pancreatic β-cell that impair insulin secretion [3]. MODY is characterized by onset before 25 years of age; patients usually lack auto-antibodies, and clinical manifestations go from slight non-ketotic hyperglycemia to severe complicated hyperglycemia [4]. Heterozygous mutations in the *glucokinase* (*GCK*) gene produce two distinct diseases, MODY type 2 (MODY2) (MIM:125851) and persistent hyperinsulinemia of infancy (MIM:601820) [5], [6]. Persistent hyperinsulinemia of infancy is associated with hyperactive GCK variants, while MODY2 is associated with GCK mutations that impair its activity [7]. GCK (hexokinase IV) catalyzes the ATP-dependent phosphorylation of glucose in the first, rate-limiting step of glycolysis in pancreatic β-cells [1]. The crystal structure determination of the enzyme by Kamata et al. [8] revealed that the enzyme exists in at least two distinct forms, the super-open ligand-free form and the closed active form that is bound to glucose and ATP. The molecule consists of two domains, namely a small and large domain separated by the glucose site. In detail, amino acid residues 1–64 and 206–439 belong to the large domain, amino acid residues 72–201 and 445–465 to the small domain, and amino acid residues 65–71, 202–205 and 440–444 to the three loops connecting the domains [8]. The GCK protein switches from an inactive conformation to a close active conformation upon ligand binding. A huge conformational transition occurs through a large rotation of the small domain [8].

The heterozygous loss-of-function GCK mutations causative of MODY2 diabetes include missense, nonsense, splicing, small deletions/insertions/duplications variants, and result in stable fasting hyperglycemia from birth (>5.5 mol/L) and rare microvascular complications [1]. Over 644 *GCK* mutations have been described, and a study of the mutational mechanisms for a number of these has shed light on GCK regulation [9].

The molecular diagnosis of MODY2 is important: to classify the type of diabetes correctly, to predict prognosis, and to initiate screening of family members [1]. It is particularly important to identify MODY2 in diabetic pregnant patients in order to start “ad hoc” treatment [10]. However, the identification of *GCK* mutations by molecular analysis will not always reveal whether a variant is really pathogenic or how serious the diabetic phenotype could be. Therefore, in this five-year study, we applied an integrated approach to investigate the effect of *GCK* mutations in the diabetic phenotype in children from southern Italy. First, we used DHPLC and mutation sequencing to identify variants, then a computational approach to predict the effect of the variants identified, and finally a functional study to determine the effect of seven candidate variants on enzyme activity and on enzyme thermo-stability.

## Results

Among the 66 enrolled patients with suspected MODY2, 28/66 were diagnosed as MODY2 based on mutations in the *GCK* gene (MODY2+) and 38/66 were MODY2-negative (MODY2−). All mutated patients were unrelated, except two pairs of siblings (patient identification: MD19/20: two sisters and MD69/70: brother/sister). The mean age at diagnosis (± Standard Deviation: SD) was lower, albeit not significantly, in MODY2+ than in MODY2− patients (105±45 months vs 113±45 months). Mean triglyceride levels did not differ between the two groups (0.6±0.2 and 0.7±0.3 mmol/L, respectively). Mean Fasting Plasma Glucose (FPG) and glycosylated hemoglobin (HbA1c) concentrations were significantly higher (*p*<0.003 and *p*<0.001, respectively) in MODY2+ than in MODY2− patients (6.7±0.8 mmol/L and 6.2±0.3% vs 6.1±0.7 mmol/L and 5.5±0.5%, respectively). The Body Mass Index zeta-score (BMIz-score) of enrolled children at first admission was always in the reference range for the children’s age (reference range: −1.5/+1.5), except in one patient (BMIz-score: 2.5). Two/28 MODY2+ patients were positive for only one type-1 diabetes auto antibody (glutamate decarboxylase: 8 and 18 U/mL). The patients were untreated until diagnosis.

### Identification of *GCK* Mutations

We identified 19 different *GCK* mutations in 28/66 patients (Table 1). Six of them were novel: two missense mutations (p.Val154Leu and p.Glu40Asp), one splice site variant (c.580-2A>T) and three deletions (p.Lys458_Cys461del, p.Arg447Glyfs, and p.Glu395_Arg397del). We also found 13 previously reported mutations: 11 missense mutations (p.Arg303Trp, p.Gly261Arg, p.Phe150Tyr, p.Ala259Thr, p.Glu70Asp, p.Lys420Glu, p.Ala188Thr, p.Tyr289Cys, p.Asp278Glu, p.Gly223Ser, and p.Ala449Thr) and two splice site variants (c.864-1G>A and c.1019+5G>A). All the detected mutations were always present in the children’s mother or father and were absent from 200 chromosomes of our euglycemic controls. Seven mutations were each present in two unrelated families: p.Arg303Trp, p.Lys458_Cys461del, p.Arg447Glyfs, p.Gly261Arg, p.Phe150Tyr, p.Lys420Glu and p.Ala259Thr, the latter was also identified in two siblings. The splice variant c.580-2A>T was present in two siblings. Table 1 shows, for each mutation (except the three splice variants c.864-1G>A, c.580-2A>T and c.1019+5G>A, the frameshift mutation p.Arg447Glyfs and the p.Lys458_Cys461del), the nucleotide position, the amino acid change (if present), the effect on the protein predicted by bioinformatics.

**Table 1:** GCK mutations detected in MODY2 children from South Italy.

Patientcode	GCKExon^a^	cDNAmutation^b^	Polyphen/SIFTprediction^c^	Aminoacid change^d^/Domainlocalization/Secondary structure	Effect on protein 3D-structure	Reference
MD01/92	8	c.907C>T	1/deleterious	p.Arg303Trp/Large domain/α8 helix	Disruption of two salt-bridges	[25]
MD05/71	10	c.1373_1385del		p.Lys458_Cys461del/Small domain/α13 helix		NOVEL
MD10	4	c.460G>T	2/tolerated	p.Val154Leu/Small domain/β-strand 6	Severe perturbation during thetransition from the super-open toclosed form. Mild local structuralalteration	NOVEL
MD12/89	10	c.1339delC		p.Arg447Glyfs/Small domain/α13 helix		NOVEL
MD16/75	7	c.781G>T	1/deleterious	p.Gly261Arg/Large domain/loop	Replacement of a small and flexiblehydrophobic residue with a largepositive residue	[26]
MD18/94	4	c.449T>A	1/deleterious	p.Phe150Tyr/Small domain/β-strand 5	Introduction of a polar residue in thehydrophobic core. Perturbation ofthe β-sheet	[27]
MD19^e^/20^e^/79	7	c.775G>A	3/deleterious	p.Ala259Thr/Large domain/loop	Influence on the hydrogen bondnetwork near the glucose-bindingcleft	[28]
MD25	2	c.210A>C	1/deleterious	p.Glu70Asp/Connection/Loopspatially near α13 helix	Weakness of salt bridge interactionwith K458 (α13 helix)	[11]
MD26/65	10	c.1258A>G	2/deleterious	p.Lys420Glu/Large domain/α12 helix	Loss of a salt bridge between K420and E440	[29]
MD38	Intron 7	c.864-1G>A		IVS7-1G>A		[9]
MD57	5	c.562G>A	1/deleterious	p.Ala188Thr/Small domain/α4 helix	Substitution of a hydrophobicresidue with a polar residue	[30]
MD59	8	c.866A>G	1/deleterious	p.Tyr289Cys/Large domain/α7 helix	Disruption of a favorable interactionbetween Y289 and M381	[9]
MD68	7	c.834G>A	1/deleterious	p.Asp278Glu/Large domain/α6 helix	Mild structural alteration	[9]
MD69^e^/70^ e^	Intron 5	c.580-2A>T		IVS5-2A>T		NOVEL
MD80	6	c.667G>A	3/deleterious	p.Gly223Ser/Large domain/β-strand 9	Perturbation of the β-strand	[31]
MD86	Intron 8	c.1019+5G>A		IVS8+5G>A		[27]
MD90	2	c.120G>C	2/tolerated	p.Glu40Asp/Large domain/α2 helix	Mild structural alteration	NOVEL
MD91	10	c.1345G>C	3/deleterious	p.Ala449Thr/Small domain in theclosed form/α13 helix	Introduction of a larger and polarside chain	[9]
MD95	9	c.1182_1191del		p.Glu395_Arg397del/Large domain/last residue of α11 helix and followingloop	Destabilization of the C-terminalregion of the helix and shorteningof loop	NOVEL

a GenBank: accession number (AH005826). ^b^The reference cDNA sequence was obtained from GenBank (NM_000162) and +1 corresponds to the A of the ATG translation initiation codon. ^c^Polyphen prediction: probably damaging (1), benign (2), possibly damaging (3). SIFT score: <0.05 deleterious variant, ≥0.05 tolerated variant. ^d^Swissprot accession number: P35557. ^e^Sibling pairs (MD19/20: two sisters; MD69/70: brother/sister).

### Bioinformatics Study of the GCK Variants

**Figure 1:**
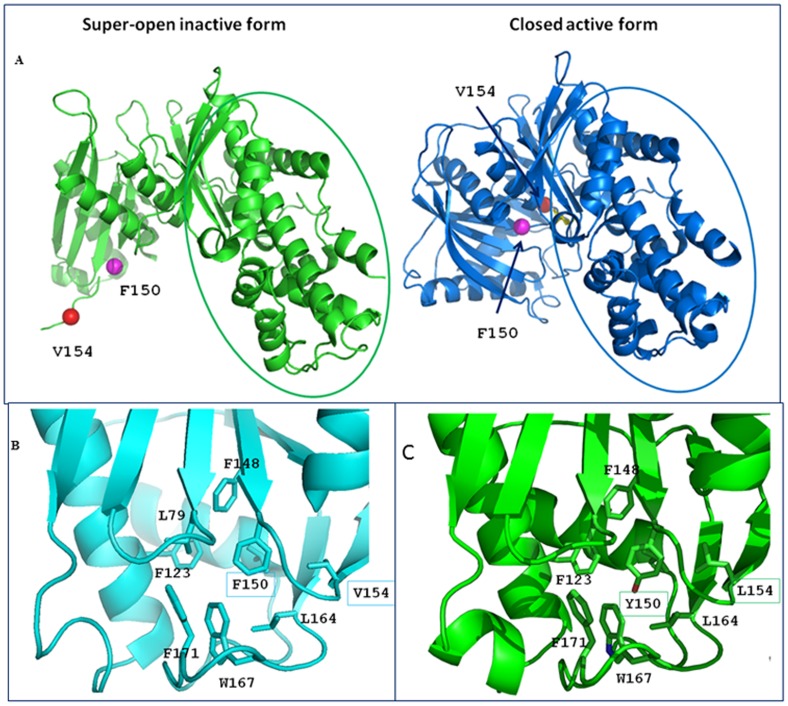
View of the mutations p.Phe150Tyr and p.Val154Leu in the whole structures and in their local environment. A : The structures of GCK in the super-open inactive form (PDB code code: 1v4t) and in the closed active form (PDB code code: 1v4s) are shown on the left and on the right, respectively. In the closed form (right) the sugar is shown as a yellow stick. In both panels, the large domain is in the same orientation and is circled. It is clear that the small domain undergoes a large conformational variation from the super-open to the closed form. Specifically the region embodying the residues Phe150 (blue sphere) and Val154 (red sphere) dramatically changes its orientation. **B**: Close-up view of the wild-type closed structure showing the hydrophobic core rich in aromatic/hydrophobic amino acids. Leu79, Phe123, Phe148, Phe150, Val154, Leu164, Trp167 and Phe171 form an intricate network of stabilizing hydrophobic interactions. **C**: Structure of GCK closed structure containing the both the Tyr150 and Leu154 mutated residues. Introduction of the oxydryl group (red) of Tyr150 within the hydrophobic core disrupts the interactions present in the wt-enzyme. The replacement of Val154 by leucine produces only small changes in the closed form.

All substitutions, except p.Val154Leu and p.Glu40Asp, were predicted to be deleterious mutations by online prediction tools (Table 1, column 4). Overall, the theoretical structural models of the mutants we obtained *in silico* (Table 1, column 5), preserve the overall protein fold. In detail, however, all mutations produced local conformational alterations − in some cases, such as p.Gly162Asp, dramatic perturbations – that well account for the functional alterations we found (Table 2). **p.Arg303Trp**-Arg303 is a highly conserved residue located in the α8 helix within the large domain. Mutation p.Arg303Trp disrupts two salt-bridges between the side chain of Arg303 and the side chain of Glu300, located in the same helix. These salt-bridges may be essential for the stability of the helix and their loss could destabilize the helix structure. **p.Val154Leu**-The p.Val154Leu mutation (Figure 1) does not cause any significant change in the local structure. Indeed, Val154 is located in the β-sheet that encompasses the small domain hydrophobic core. Its substitution with a leucine residue does not affect the hydrophobic interactions. Nevertheless, Val154 is involved in the large conformational variation from the super-open to close form upon glucose binding (Figure 1A). **p.Gly261Arg**-Gly261 is located in the loop connecting the β-sheet and the α6 helix in the large domain. p.Gly261Arg is a dramatic mutation because the small, flexible hydrophobic Gly residue is replaced by a very large Arg residue that bears a positive net charge. This substitution causes a local re-arrangement that involves the nearby residues such as Leu266 and Leu270. This process results in destabilization of the local structure.

**Table 2:** Kinetic constants of human recombinant wild type-GCK and mutant β-cell GST-GCK fusion proteins.

Preparation	Protein yield(mg/L of culture)	Kcat (s^−1^)	S_0.5_ for glucose (mmol/L)	nH	Km for ATP(mmol/L)	I_a_	
Wild-type GST-GCK	3.12±0.91	44.10±7.50	7.20±0.40	1.40±0.08	0.40±0.00	1.0±0.07	
GST-GCK(Glu70Asp]	2.35±0.55	27.40±4.80*	20.10±3.20†	1.20±0.07	0.25±0.05	0.25±0.06**	
GST-GCK(His137Asp]	2.82±0.47	17.20±1.20**	18.10±3.10*	1.10±0.03**	0.23±0.03*	0.26±0.09**	
GST-GCK(Phe150Tyr]	3.07±0.55	9.92±2.38*	101.40±6.20**	1.06±0.05*	3.11±0.48*	0.014±0.003*	
GST-GCK(Val154Leu]	2.77±0.48	46.60±4.40	26.00±3.40*	1.54±0.01	1.62±0.16*	0.099±0.036**	
GST-GCK(Gly162Asp]	2.78±0.58	Undetectable activity					
GST-GCK(Arg303Trp]	1.58±0.26	14.60±1.38**	4.62±0.10**	1.52±0.02	0.29±0.01*		0.59±0.09*
GST-GCK(Arg392Ser]	2.44±0.74	41.30±11.30	11.90±1.70*	1.30±0.03	0.63±0.03*		0.60±0.25

Data represent means ± SEM of 3 separate enzyme expressions each tested in duplicate. Note that the Hill coefficient (nH) and the relative activity index (I_a_) are unit less. Kcat: GCK catalytic constant; S_0.5_: affinity constant for glucose; nH: Hill coefficient; Km for ATP: affinity constant for ATP; I_a_: GCK activity index. (*)*p*<0.05, *t* test; (**)*p*<0.005, *t* test.

**p.Phe150Tyr**-Phe150 is a highly conserved residue located in the β-sheet that encompasses the small domain hydrophobic core. The p.Phe150Tyr mutation (Figure 1B, C) introduces a polar residue inside the hydrophobic core in a region rich in phenylalanine. This replacement can influence the stability of the β-sheet thereby altering the structure of the domain. **p.Ala259Thr-**Ala259 residue is located in the large domain near the glucose-binding cleft. Introduction of a larger and polar side chain of threonine in the p.Ala259Thr mutant could influence the hydrogen bond network in that area. In fact, Thr259 can compete with other residues in the formation of hydrogen bonds with two water molecules. See our previous study for a description of the **p.Glu70Asp** variant [11]. **p.Lys420Glu-**Mutation p.Lys420Glu involves an inversion from a positively charged lysine to a negatively charged glutamic acid. The substitution affects the interaction of Lys420 with surrounding residues. Indeed, Lys420 is located in the α12 helix (Figure S1) in the large domain and forms a salt bridge with Glu440 located in a connecting loop between the two domains. The loss of this bond, caused by the mutation, could destabilize the region. **p.Ala188Thr-**The p.Ala188Thr mutation alters a highly conserved amino acid. Ala188 is located in the α4 helix within the small domain. The mutation leads to the substitution of a hydrophobic residue, alanine, with a polar residue, tyrosine, on the hydrophobic side of an amphipathic helix. Thr188 can form hydrogen bonds through the hydroxyl group with the side chains of the Ser127 and Asp124 residues on the α3 helix that is on the surface of the enzyme. The introduction of the threonine can cause a different arrangement of such side chain. **p.Tyr289Cys-**Tyr289 is located in the α7 helix in the large domain. The substitution of the bulk tyrosine in position 289 with the smaller cysteine side chain leads to the formation of a cavity. The mutant disrupts a favorable interaction between Tyr289 and Met381 of the α11 helix that may be important to keep helices together. It is noteworthy that, in our model, Cys289 is not bound to the nearby Cys230 because the distance (4.1 Å) between the two sulfur atoms too long for a disulfide bond formation. **p.Asp278Glu-**The p.Asp278Glu mutation affects a highly conserved amino acid. Asp278 is located on the polar side of the α6 helix in the large domain. The replacement of Asp with a Glu residue in the mutant does not seem to cause significant changes in the enzyme structure. **p.Gly223Ser-**Gly223 is a highly conserved residue located in the β-sheet of the large domain hydrophobic core. The substitution with a serine involves the serine side-chain hydrogen bonding to Cys 233, close to Gly223 in the structure of GCK and could perturb the β-strand. **p.Glu40Asp-**The pGlu40Asp mutation substitutes a conserved glutamate residue with aspartate. Glu40 is located on the polar side of the α2 helix in the large domain. These amino acids are acidic residues but the side chain of the aspartate is shorter than the side chain of the glutamate. Nevertheless, the mutation does not seem to cause significant changes in the structure of the enzyme. **p.Ala449Thr-**Ala449 is a highly conserved residue located on the C-terminal α13 helix. This helix is part of the small domain in the closed form, but it lies between the two domains in the super-open form. In the closed form, the small domain has a three-layer architecture and the α13 helix is in the inner layer. At the domain interface, the α13 helix makes favorable interactions with the α5 helix of the large domain. Because the conformational features of this region are essential for the super-open (inactive form)/closed (active form) conversion, the introduction of a larger and polar side chain of the threonine in the p.Ala449Thr mutant could influence the enzymatic activity and/or stability. **p.Glu395_Arg397del–**The p.Glu395_Arg397 deletion causes the elimination of the last residue, Glu395, of the α11 helix in the large domain and two residues of the following loop, Ser396 and Arg397. In the wild-type enzyme, Glu395 is involved, through the backbone N atom, in the formation of a hydrogen bond of the α11 helix. Its deletion in the mutant causes the lack of this bond and could destabilize the C-terminal region of the helix. Moreover, also the backbone N atom of Arg397 interacts with an oxygen atom of Arg394 thereby stabilizing the helix. In the mutant, the loop becomes shorter and the side chain of Arg394, which in the wild-type enzyme is directed towards the loop, changes the orientation because of steric interactions. This change disrupts interactions between the side chain of Arg394 and some residues of the loop. Although the interaction between the side chains of Arg394 and Ser433 in the loop following the α12 helix is conserved, the binding appears to be weakened compared with the wild-type enzyme.

### Kinetic Analysis and Thermo-stability of Recombinant GCK Mutants

**Figure 2:**
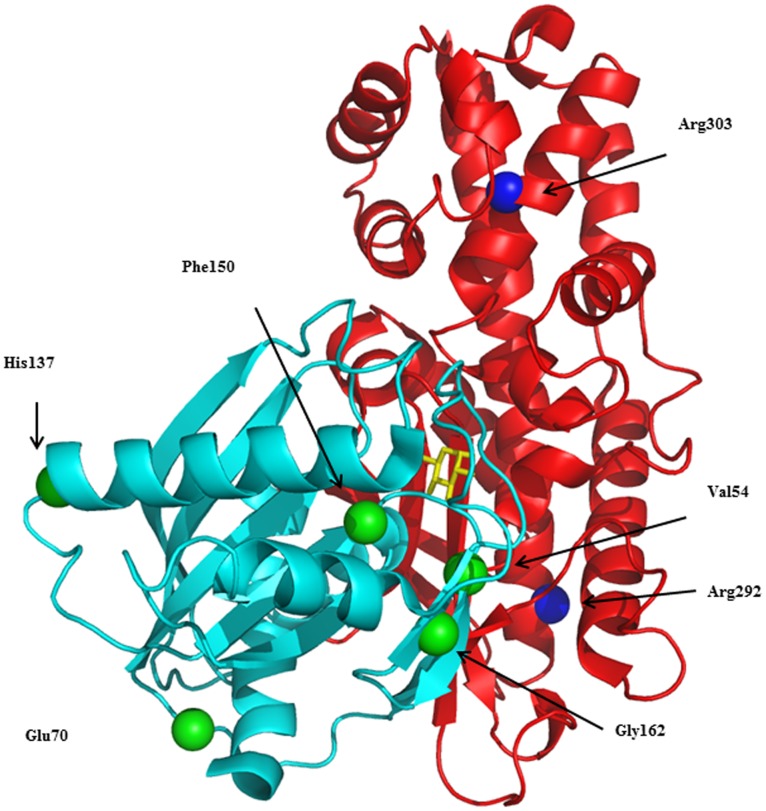
Distribution of the selected GCK mutations. The structure of GCK in the active closed form is shown (PDB code: 1v4s); the small and large domains are drawn in cyan and red, respectively; glucose appears as a yellow stick. Mutation sites are shown as green or blue spheres.

**Figure 3:**
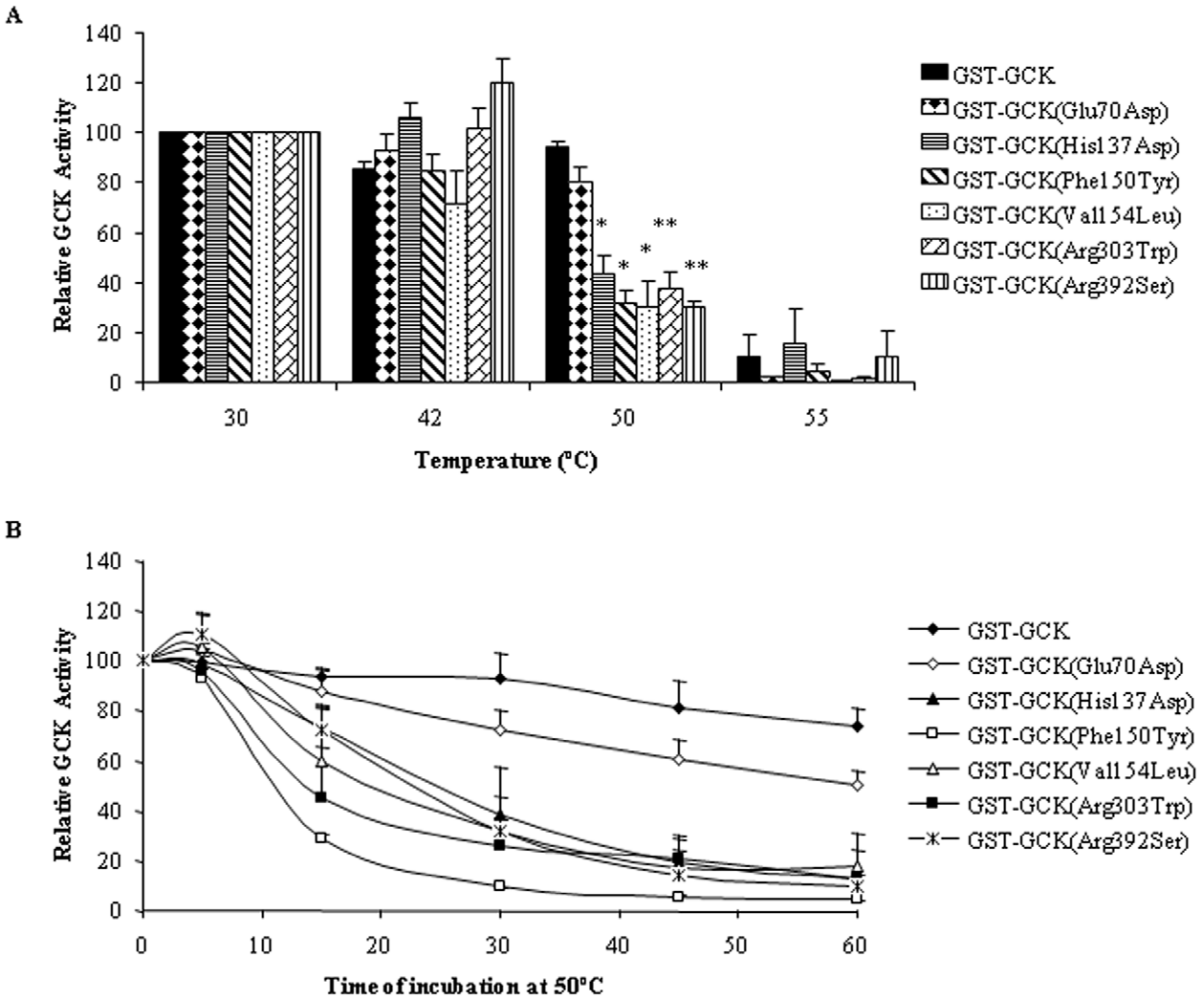
Effect of temperature on the stability of GST-GCK mutants . Stock enzyme solutions were diluted to 250 µg/ml in storage buffer containing 30% glycerol, 50 mM glucose, 10 mM glutathione, 5 mM DTT, 200 mM KCl and 50 mMTris/HCl, pH 8.0. Panel A: The enzyme solutions were incubated for 30 min at temperatures ranging from 30 to 55°C and then assayed at 30°C as described in the Methods section. Panel B: The enzyme solutions were incubated for periods of time from 5 to 60 min at 50°C. Results are means and SEM of three independent enzyme preparations for each mutant except for GST-GCK (Phe150Tyr) which corresponds to two independent enzyme preparations. (*) *p*≤0.03, (†) *p*<0.008.

To select representative mutations for a functional study to evaluate their pathogenic significance, we considered all the mutations found in our population in the last decade (11 and present work) in terms of severity and location. All the selected mutations were mapped in the whole GCK structure: p.His137Asp, pPhe150Tyr, p.Val154Leu, p.Gly162Asp, in the small domain, Arg303Trp and p.Arg392Ser, in the large domain and p.Glu70Asp in a loop connecting the two domains. Three among these mutations, (p.Gly162Asp, p.His137Asp and p.Arg392Ser) were the most severe and none have been produced or characterized. Figure 2 shows the position of selected mutated residues in the structure model of the GCK enzyme. All variants displayed reduced enzyme activity versus the wild-type GST-GCK, as shown by the I_a_ index. The kinetic characteristics of GST-GCK-wt and GST-GCK-mutants, determined *in vitro* enzymatic assays, are shown in Table 2. Mutations p.Gly162Asp, p.Phe150Tyr and p.Val154Leu, localized in the core of the molecule, not far from the substrate binding site, produced the strongest effect. The p.Gly162Asp change is very deleterious since no enzymatic activity was detected in the mutant protein, and mutations p.Phe150Tyr and p.Val154Leu reduced enzyme activity to below 10% of the wild-type enzyme. In contrast, mutations p.Glu70Asp, p.His137Asp, p.Arg303Trp and p.Arg392Ser, localized in more peripheral positions, retained at least 25% of wild-type activity. We also evaluated the protein stability of wild-type and of mutant GST-GCK and the time-course of thermal inactivation at different temperatures (Figure 3A and B respectively). Wild-type GCK activity remained practically constant under temperatures up to 50°C, but fell abruptly at 55°C. In contrast, the enzyme activity of all mutants, except GST-GCK (p.Glu70Asp), rapidly decreased by more than 50% at 50°C (Figure 3A). The time-course analysis of GCK thermal inactivation indicated that the mutants rendered the enzyme thermo-unstable (more than 50% of GCK activity was lost within 30 min at 50°C), whereas 50% of wild-type GCK activity was present after 30 min of incubation at the critical temperature (Figure 3B).

## Discussion

A correct diagnostic approach to the MODY2 patient is important to avoid useless, expensive analyses and misclassification of the diabetes. In this study, we characterized the *GCK* gene by DHPLC followed by sequence analysis in 66 patients with suspected MODY2 enrolled between 2006 and 2010. We identified 19 GCK mutations in 28 children accounting for 42.4% of suspected MODY2 children. Our data are similar to those we obtained between 2001 and 2005 [11] and are also in agreement with the high prevalence of MODY2 (up to 61% of MODY forms) found in Italy and in southern Europe [12], [13]. Among the 19 GCK variants described herein, six are new (p.Lys458_Cys461del, p.Val154Leu, p.Arg447Glyfs, IVS5-2A>T, p.Glu40Asp and p.Glu395_Arg397del) and 13 previously reported. Glucokinase missense mutations are the most frequent causes of MODY2 [9]. In our study, the missense mutations (13/19: two new and 11 known), and deletions (n  = 3) were distributed throughout the protein: six/16 (38%) in the small domain, nine/16 (56%) in the large domain and one/16 (6%) in the connecting region of the protein. These findings are in agreement with a study in which no hot spot mutations were reported [9].

In the attempt to understand how the detected mutations could contribute to MODY2 insurgence, we searched for structure/function correlations of the disease-causing mutated proteins. First, we evaluated the impact of each mutation (except splice variants and deletions) on the enzyme 3D-structure and then, for seven selected mutations, we carried out functional studies. All the mutations considered here are buried in the enzyme, except for p.Glu70Asp that is located on the surface of the protein, and all mutations are far from the active and ATP binding sites (Table 1). Apart from p.Val154Leu and p.Phe150Tyr that undergo large movements during the conformational transition from the super-open to the closed active form, all the mutant structures exhibit local structural alterations (Table 1) that well correlate to kinetic parameters and thermal inactivation data (Table 2, Figure 3). In all cases, the amino-acid replacement either provokes the loss of stabilizing interactions or generates unfavorable interactions that may destabilize the enzyme.

We evaluated the effects on GCK kinetics and thermo-stability of seven GCK variants distributed in different functional domains of the enzyme: three variants described in the present study (p.Phe150Tyr, p.Val154Leu, p.Arg303Trp) and four (p.Glu70Asp, p.His137Asp, p.Gly162Asp and p.Arg392Ser) previously reported [11]. The GST-GCK (p.Glu70Asp) mutant showed a GCK activity index significantly lower than the wild type (mean I_a_ = 0.25). This effect is due to discrete defects in the kinetic constants, in particular, glucose affinity was significantly reduced (S_0.5_ for glucose: 20.1±3.2 vs 7.2±0.4 mM). Variant p.Glu70Lys displayed a similarly decreased activity index (mean I_a_  = 0.31) without significant thermo-instability [14], [15]. The substitution of histidine with aspartic acid (p.His137Asp) affected GCK function (mean I_a_  = 0.26). As shown by molecular modelling, this mutation introduces a negative charge in the region that may affect the charge distribution of the protein [11]. The previously reported mutation p.His137Arg, in which His is replaced by the positively charged arginine, did not affect enzyme activity (mean I_a_  = 0.92) [15]. This finding shows that a negatively charged amino acid is not tolerated at this site. p.Phe150Tyr and the novel p.Val154Leu mutations dramatically reduced GCK activity (mean I_a_  = 0.014 and 0.099, respectively) and thermo-stability (<40% at 50°C/30 min than in wild-type). Both mutations displayed high S_0.5_ and ATP Km values, which indicate a greatly reduced affinity for glucose and ATP. In particular, the cooperativity of p.Phe150Tyr was significantly reduced (nH  = 1.06 vs 1.40, *t* test, *p*  = 0.038). It is noteworthy that Val154 is one of residues that undergo large movements during the transition from the super-open to the closed form. Even though the replacement of Val by Leu provokes only small local perturbations, the latter may be relevant during the conformational transition induced by ATP and glucose binding. The substitution of glycine 162 by aspartic acid (p.Gly162Asp) affected the hydrophobic core of the enzyme and, as predicted by molecular modelling [11], markedly alters the structure and dynamics of the domain. This is one of the most dramatic mutations we identified as it introduces a net negative charge into a hydrophobic environment [11]. In fact, the enzyme activity of this mutant was undetectable. Although any reduction in the I_a_ below 30% of the wild type would have little further effect on the fasting plasma glucose [15], it is noteworthy that fasting glycemia was higher in the child with this mutation (10.0 mmol/L) [11] than in other MODY2 children (mean 6.7±0.8 mmol/L). Arg 303 is located in the α8 helix and molecular modelling indicates that the Arg-to-Trp change at this position would destabilize the helix structure. Mutation p.Arg303Trp caused a reduction of the activity index (mean I_a_  = 0.59), mainly due to a decrease in the catalytic constant, being both glucose and ATP affinities modified. Our results concord with the finding that other mutations in the same helix (p.Leu309Pro and p.Arg308Trp) increased thermal inactivation and/or modified glucose affinity [16], [17]. Taken together, these results suggest that the α8 helix plays a role in the regulation of substrate affinity and protein stability. Mutation p.Arg392Ser is located in the protein periphery and, like the neighboring mutation p.Arg397Leu [17], it only slightly affected GCK activity. Thus, our mutants reduced the enzyme’s catalytic activity by altering one or more kinetic parameters. Moreover, all mutants, except GCK-Glu70Asp, displayed thermal-instability, which has been implicated in hyperglycemia in MODY2 patients [17], [18]. Nevertheless, additional defects caused by these mutations on other mechanisms of GCK control, such as post-translational regulation, interaction with other protein partners or organelles, cannot be excluded. Globally our evaluation of enzyme activity indicates that all seven mutants play a pathogenic role in MODY2 insurgence. In addition although altered glucose and ATP binding, and thermal stability appear to be the major causes of the disease, in a few cases mutations affected cooperativity and molecular motions, and hence impaired enzyme activity.

Although the in vitro functional evaluation of a GCK-mutant is a useful method with which to predict the effect exerted by a DNA change on enzyme activity, it is not a practical approach to the diagnosis of MODY. In our experience, taking into account all the experimental data concerning the seven mutants expressed, we found that the mutations induced structural alterations predicted by modeling that were in good agreement with kinetics/thermostability analyses. Therefore, this approach could be a reliable surrogate to predict the pathogenic role of a GCK variant.

In conclusion, in this five-year update of GCK mutations in MODY2 children from southern Italy, we have identified six new GCK variants thereby expanding the MODY2 mutation panel. Furthermore, our study, carried out by integrating DHPLC, sequencing, bioinformatics and functional analysis, provides new information about the structure-function relationship of human glucokinase mutations and MODY2.

## Materials and Methods

### Ethics Approval

The study was conducted according to the Helsinki II declaration and it was approved by the Ethics Committee of the School of Medicine Federico II, Naples, Italy.

Written informed consent to the study was obtained from each adult subject and from both parents of children.

### Subjects

Between 2006 and 2010, 720 hyperglycemic children were monitored at the Departments of Pediatrics of the University of Naples “Federico II” and of the Second University of Naples, Italy. Sixty-six patients (mean age 109 months, 53% girls) who had fasting hyperglycemia (>5.5 mmol/L), HbA1c <7.0% and a family history of diabetes for at least two consecutive generations were enrolled in the study as suspected MODY2 individuals. The autoimmune markers of type-1 diabetes (glutamate decarboxylase, protein tyrosine phosphatase-like protein and insulin antibodies) were evaluated; the presence of more than one antibody was considered an exclusion criterion. One hundred unrelated euglycemic controls (mean age 363 months, 69% girls) were recruited at CEINGE (Advanced Biotechnologies, s.c.a.r.l. Naples) also between 2006–2010.

Patients, their mother and father, and controls provided 2 blood samples for biochemical testing and for DNA extraction. BMIz-score (Center for Disease Control normative, http://www.cdc.gov), family history of diabetes and/or other diseases, birth weight and age at admission were recorded for each patient. FPG, triglycerides (evaluated with routine methods) and HbA1c measure with HPLC (HLC-723 G7 TOSOH Bioscience Tokyo, Japan) were evaluated in each suspected MODY2 child. Genomic DNA from patients, their mother and father, and controls was extracted with the Nucleon BACC 2 kit (Amersham Biosciences Europe, Milan, Italy).

### *GCK* Gene Analysis

The 10 exons, including their flanking intronic regions, of the *GCK* gene were amplified using primers and PCR conditions described elsewhere [11]. The amplified fragments were denatured at 95°C for 10 min and then renatured for 10 min to generate heteroduplices. Each fragment was run on the DHPLC WAVE DNA fragment analysis system (Transgenomic, Inc. Omaha, NE) using DHPLC conditions reported in Table S1. Any additional or abnormal peak shape observed in the chromatogram was further sequenced (ABI PRISM 3730; Biosystems Foster City, CA, USA). All sequences were analysed in comparison with the wild-type reference sequence (NM_000162, http://www.ncbi.nlm.nih.gov/nuccore/NM_000162) by the ABI Seqscape software v.2.5 (Applied Biosystems). The mutations and variants were then numbered according to the Human Genome Variation Society (http://www.hgvs.org/).

### Analysis of *GCK* Mutations

We first evaluated each detected GCK mutation by bioinformatics (Polyphen and SIFT algorithms and Modeller programs). We also examined the enzyme function of seven mutations among the most severe found in our population in the last decade and never characterized. The mutations for the latter study mapped in the whole enzyme structure (small, large and loops connecting domains).

### Bioinformatic Analysis

Two free online prediction programs, Polyphen (http://genetics.bwh.harvard.edu/pph/) and SIFT (http://sift.jcvi.org/www/SIFT_seq_submit2.html), [1] were used to predict the effect of GCK mutations on the protein. The Polyphen program [19] is a tool for the prediction of the impact of an amino acid substitution on the structure of a human protein using straight forward physical and comparative considerations. The SIFT program [20] tests a query sequence and uses multiple alignment information to predict tolerated and deleterious substitutions for every position of the query sequence. SIFT is a multistep procedure that searches for similar sequences, chooses closely related sequences that may share a function similar to that of the query sequence, obtains the alignment of these chosen sequences, and calculates normalized probabilities for all possible substitutions from the alignment. Models of all mutants of GCK were generated *in silico* with the Modeller 9v8 program using the active form of the GCK crystal structure as template [2.3 Å, Brookhaven Protein Database code (PDB code) 1v4s]. The inactive super-open form (3.4 Å, PDB code 1v4t) was also considered for comparison.

After alignment with using BODIL [21], the query and template sequences were used as input in the Modeller program [22] and 20 models were generated. The most abundant conformers of the replaced residue were selected and a simulated annealing procedure was carried out to optimize side chain conformations. The model that presented the best Modeller Discrete Optimized Protein Energy (DOPE) score was selected to be used in subsequent analyses. The initial models of selected mutants were energy minimized with the GROningen MAchine for Chemical Simulations (GROMACS) package using the GROningen MOlecular Simulation (GROMOS) 43a1 force field, to regularize the protein structure geometry. The molecules were solvated in boxes containing simple point charge water molecules. The energy minimization was obtained by 1000 steps of the steepest descent method and subsequently by 1000 steps of the conjugate gradient method. The notation used for secondary structure was taken from Kamata et al. [8].

### Production of Recombinant Wild-type and Mutant Glutathionyl-S-transferase-glucokinase (GST-GCK)

Recombinant human wild-type beta cell GCK fused to GST (GST-GCK) was prepared as described previously [23]. Mutations were introduced into the GST-GCK construct by PCR using the QuikChange® II Site Directed Mutagenesis Kit (Stratagene, La Jolla, CA, USA); the oligonucleotides are reported in Table S2. Mutant constructs were checked by sequencing and digestion with specific restriction enzymes (Table S2). Fusion proteins from *E. coli* were expressed and purified as described previously [24]. Fusion proteins were stored at a concentration of about 1 mg/ml at –80°C in 30% glycerol, 50 mM glucose, 10 mM glutathione, 5 mM DTT, 200 mM KCl and 50 mM Tris/HCl, pH  = 8.0. Protein concentrations were determined with the Bio-Rad Protein Assay (Bio-Rad Laboratories, GmbH München Germany), and bovine serum albumin as standard. GST-GCK purification was verified by Coomassie-stained SDS-PAGE as a single band of 75 kDa.

### Enzymatic Assay

GCK activity was measured spectrophotometrically on a UVIKONxl spectrophotometer (Secoman, France), using a NADP^+^-coupled assay with glucose-6-phosphate dehydrogenase. Determination of kinetic parameters and thermal inactivation were performed as previously described [23]. The Km for ATP was measured at a glucose concentration near the relative S_0.5_ values (7, 15, 8.5, 100, 20, 5 and 11 mM respectively for wild type, p.Glu70Asp, p.His137Asp, p.Phe150Tyr, p.Val154Leu, p.Gly162Asp, p.Arg303Trp and p.Arg392Ser). Thermal inactivation experiments were assayed at a glucose concentration of 100 mM. Results are reported as means ± standard error of the mean (SEM) of three independent enzyme preparations assayed at least in duplicate.

### Statistical Analysis

Variables are reported as mean±SD (continuous variables) or mean ±SEM (categorical variables). We used the Student *t* test to compare variables; significance was set at *p*<0.05. The SPSS statistical software was used.

## Supporting Information

Figure S1 **Close-up view of the p.Lys420Glu mutation at the inter-domain interface.** The small and large domains are drawn in cyan and red, respectively. Helix 13 is shown in orange. Lys420 (red stick) forms a salt-bridge with Glu440 (yellow stick) which is located in a loop connecting the two domains.
(DOCX)

Table S1 **DHPLC conditions to detect GCK variants.**
(DOCX)

Table S2 **Primers used for site-directed mutagenesis in GCK cDNA.**
(DOCX)
