# Supplementary figures and images for: Glucokinase (GCK) Mutations and Their Characterization in MODY2 Children of Southern Italy
Source: PLoS One. 2012 Jun 20;7(6):e38906. doi: 10.1371/journal.pone.0038906 (PMC3385652; doi:10.1371/journal.pone.0038906)

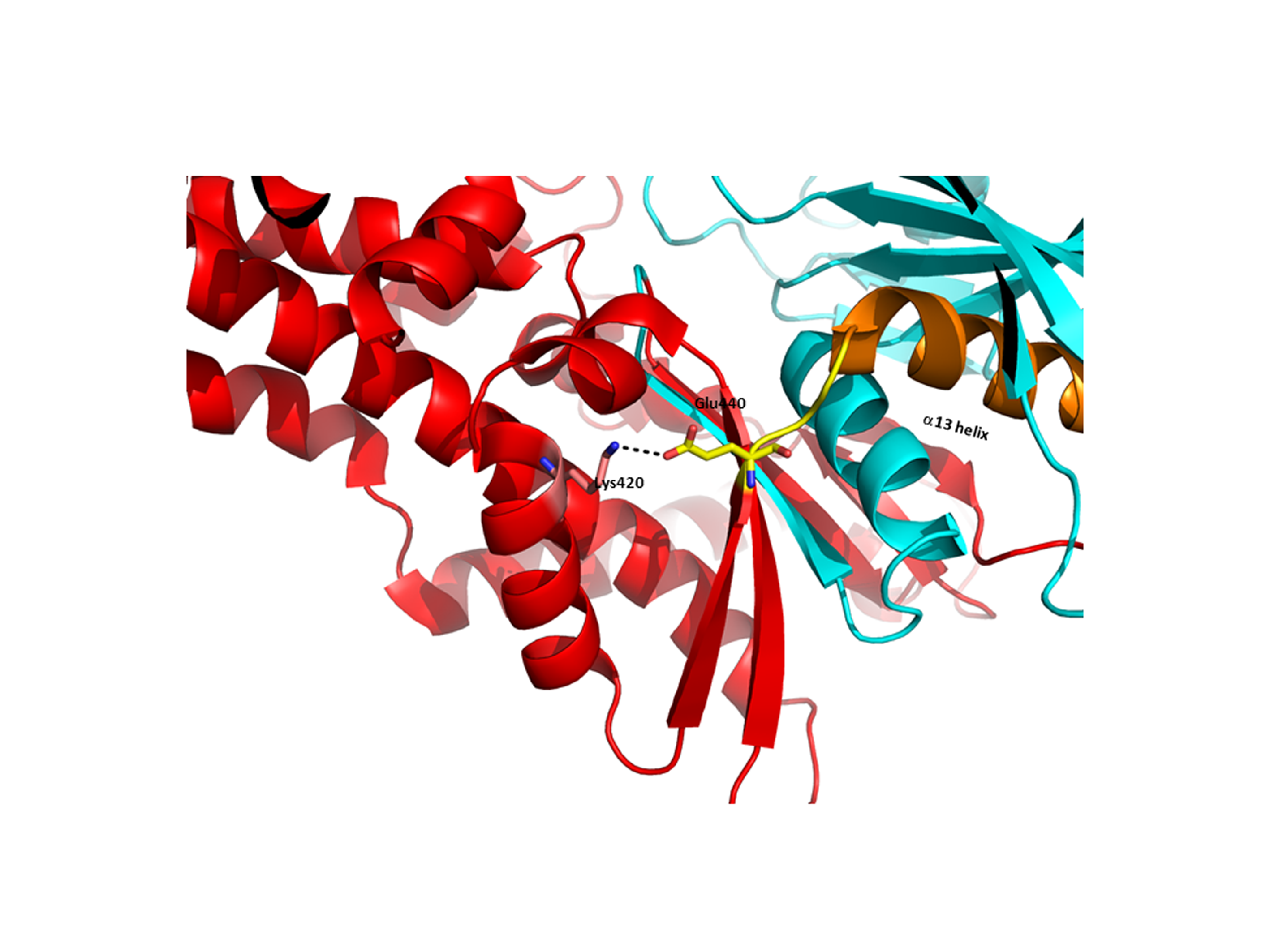

Supplement: Figure S1 — Close-up view of the p.Lys420Glu mutation at the inter-domain interface. The small and large domains are drawn in cyan and red, respectively. Helix 13 is shown in orange. Lys420 (red stick) forms a salt-bridge with Glu440 (yellow stick) which is located in a loop connecting the two domains. (DOCX) [file pone.0038906.s001.docx]
