# Supplementary material for: Glucokinase (GCK) Mutations and Their Characterization in MODY2 Children of Southern Italy
Source: PLoS One. 2012 Jun 20;7(6):e38906. doi: 10.1371/journal.pone.0038906 (PMC3385652; doi:10.1371/journal.pone.0038906)
Supplement: Table S1 — DHPLC conditions to detect GCK variants. (DOCX) [file pone.0038906.s002.docx]

**Table S1.** DHPLC conditions to detect GCK variants**.**

| **EXON** | **ELUTION TEMPERATURE (°C)** |
| --- | --- |
| 1a | 60.5-62.5-64 |
| 2 | 63.5 |
| 3 | 63- 63,6 |
| 4 | 62- 62.8 |
| 5+6 | 61.8 |
| 7 | 64.3- 65 |
| 8 | 63- 64- 65 |
| 9 | 64.5- 65.5- 66.6 |
| 10 | 63.5- 65 |
