# Supplementary material for: Glucokinase (GCK) Mutations and Their Characterization in MODY2 Children of Southern Italy
Source: PLoS One. 2012 Jun 20;7(6):e38906. doi: 10.1371/journal.pone.0038906 (PMC3385652; doi:10.1371/journal.pone.0038906)
Supplement: Table S2 — Primers used for site-directed mutagenesis in GCK cDNA. (DOCX) [file pone.0038906.s003.docx]

| **Mutated residue** | **Primer sequence^*^** | **Restriction enzyme** |
| --- | --- | --- |
| Glu70Asp | 5’-cgc tcc acc cca gaa gg**a** tc**c** ga**t** gtc ggg gac ttc ct-3’ | *BamHI* |
| His137Asp | 5’-c gac ttc ctg gac aag **g**at cag atg aaa cac aag aag ct**t** ccc ctg ggc-3’ | *HindIII* |
| Arg392Ser | 5’-g gcg ggc gtc at**t** aa**t** **a**gc atg cgc gag agc cgc-3’ | *AseI* |
| Gly162Asp | 5’-g agg cac gaa gac atc ga**t** aag g**a**c atc ctt ctc aac tg-3’ | *ClaI* |
| Val154Leu | 5’-gc ttc acc ttc tcc ttt cct **t**tg agg cac gaa ga**t** atc gat aag g-3’ | *EcoRV* |
| Arg303Trp | 5’-ggc gag ctc gtg **t**gg ct**a** gtg ctg c-3' | *BfaI* |
| Phe150Tyr | 5’-cac aag aag ct**g** ccc ctg ggc ttc acc t**a**c tcc ttt cc-3’ | *HindIII* |

**Table S2.** Primers used for site-directed mutagenesis in GCK cDNA

*^*^*The reference GCK cDNA sequence was obtained from Genbank (NM_000162, <http://www.ncbi.nlm.nih.gov/nuccore/NM_000162>) and +1 corresponds to the A of the ATG translation initiation codon. The restriction sites are underlined and the point mutations are in bold.
